# Sentiment Analysis of Shared Tweets on Global Warming on Twitter with Data Mining Methods: A Case Study on Turkish Language

**DOI:** 10.1155/2020/1904172

**Published:** 2020-09-07

**Authors:** Yasin Kirelli, Seher Arslankaya

**Affiliations:** Sakarya University, Engineering Faculty, Industrial Engineering Department, Sakarya, Turkey

## Abstract

As the usage of social media has increased, the size of shared data has instantly surged and this has been an important source of research for environmental issues as it has been with popular topics. Sentiment analysis has been used to determine people's sensitivity and behavior in environmental issues. However, the analysis of Turkish texts has not been investigated much in literature. In this article, sentiment analysis of Turkish tweets about global warming and climate change is determined by machine learning methods. In this regard, by using algorithms that are determined by supervised methods (linear classifiers and probabilistic classifiers) with trained thirty thousand randomly selected Turkish tweets, sentiment intensity (positive, negative, and neutral) has been detected and algorithm performance ratios have been compared. This study also provides benchmarking results for future sentiment analysis studies on Turkish texts.

## 1. Introduction

Downpour, storm, rising temperatures, sea level, and retreating glaciers are considered as the main headlines among the indicators of climate change [1–6]. Thanks to the popularity of Twitter and easily accessible Application Program Interface (API) [7–9], tweets can be stored by topics related to hashtags. In addition to academic researchers, many firms pay attention to Twitter mainly because of commercial purposes. These firms also use Twitter to interact with their investors and customers. Comparatively to the traditional media, Twitter's impact is obvious. However, to take advantage of Twitter data, firms require to store and analyze these substantial data produced by Twitter daily. In 2018, more than 336 million active users tweet more than 500 million times per day [10].

Social media and especially Twitter are getting more popular, and its domain becomes stronger than traditional media tools. More users in the social media means more data to access. For this reason, data-based applications like disaster detection, election predictions, information filtering, and influencing opinions make use of this trend. One of these is sentiment analysis [11–13], which is one of the most attractive fields [14].

Sentiment analysis [15, 16] is based on the language of a text, and modeling is established by a text from the same language. Because of that, in literature text, analysis of Turkish is limited, and mostly, it is emphasized in English. Due to word structure of Turkish being different from English, the analysis is more complicated.

Machine learning methods [12, 17–19] have been commonly used in emotion analysis problems in previous studies. Pang et al. work on compared several machine learning methods to determine the characteristics of emotions [20]. Kaur et al. present that support vector machine (SVM) is used as a hybrid method to analyze emotion on English Twitter data [21]. Taboada et al. have been worked on a label assignment process, which reflects a positive or negative emotion has been used by using a dictionary-based approach [22]. In [23], in the use of Turkish-based approach on the study of artificial neural networks, support vector machines, Naive Bayes, and K-NN neighbors using various machine learning methods have been compared by the results. Since the dictionary-based approach studies are not enough for the studies of Turkish sentiment analysis, the dictionary has been formed by various methods. According to the results of studies, it has been seen that the emotion analysis studies conducted on Turkish texts are relatively low and insufficient compared to the studies which were conducted with English texts. Since the structure of the Turkish language is different according to the English language, an approach to the Turkish language needs to be developed in order to achieve a high success in the sentiment analysis. In this study, we aimed to compare the effect of different quality selection methods on the performance of classification in the sentiment analysis on Turkish Twitter posts. Unlike other similar studies, an integrated classification method is recommended. Additionally, Turkish NLP library has been used differently to reduce the number of features.

In this study, emotional textual analysis is implemented regarding the sensibility of society towards climate change, one of the most important environmental threats. In the first part, data collection is processed. Feature selection for modeling is described in Section 3. Sentiment analysis models are described in Section 4. In the final section, classification and conclusive comparisons take place.

## 2. Materials and Methods

Generally, classifiers can be categorized in many ways, namely, with being supervised or unsupervised. In order to test different methodologies, different classifiers belonging to relatively different realms of classification are chosen, namely, Naïve Bayesian, K-NN (nearest neighbor), and support vector machine (SVM).

### 2.1. Naive Bayesian

As mentioned in previous chapters, Weka Software is used in all analyses. Naïve Bayes classifier in Weka uses probabilistic Naïve Bayes classifier, which is used as descriptive and complementary classifier algorithm, mainly making use of Bayes rule, shown as follows:

(1) $$\begin{array}{c} \arg_{Y}^{\max}=P\left(Y\left|X_{1},X_{2}\ldots X_{n}\right.\right), \end{array}$$

(2) $$\begin{array}{c} P\left(\left.Y\right|X_{1},X_{2}\ldots X_{n}\right)=\frac{P\left(\left.X_{1},X_{2}\ldots X_{n}\right|Y\right).P\left(Y\right)}{P\left(X_{1},X_{2}\ldots X_{n}\right)}. \end{array}$$

Naïve Bayes is based on learning from data, and it means that, in order to learn model occurrence of every output calculated, it is named as prior (second term of nominator in equation (2)). Likelihood probability (first term of nominator in equation (2)) is then calculated and multiplied and divided by normalization constant (denominator term in equation (2)).

### 2.2. K-NN (Nearest Neighbor)

In pattern recognition, the K-nearest neighbor algorithm (or K-NN for short) is a nonparametric method used for classification and regression. It is based on the idea that instance must be in a close distance when compared to its closest neighbors [24].

### 2.3. SVM (Support Vector Machine)

SVM algorithm is a supervised learning algorithm and binary classifier [25]. It is mostly used to solve classification problems [26]. Support vector machine (SVM) is used to separate data belonging to two classes in a most suitable way; to implement this procedure, hyperplanes are specified [27].

## 3. Proposed System

In this section, data preparation process is explained before classification. Sentiment analysis through texts is classified using Turkish language. Therefore, Turkish tweets are taken with hashtag-related global warming on Twitter. In the next section, the word roots of the sentences are found, and data pollution is reduced.

### 3.1. Data Collection

Twitter API (Application Programming Interface), like the other APIs, is an independent platform gathered to the developers which is separate from the main website accessed by the main users. The platform sends the JSON (Java Script Object Notation) response value. JSON response value consists of tweet object, user information, text of the tweet, upload date, and location data.

**Figure 1:**
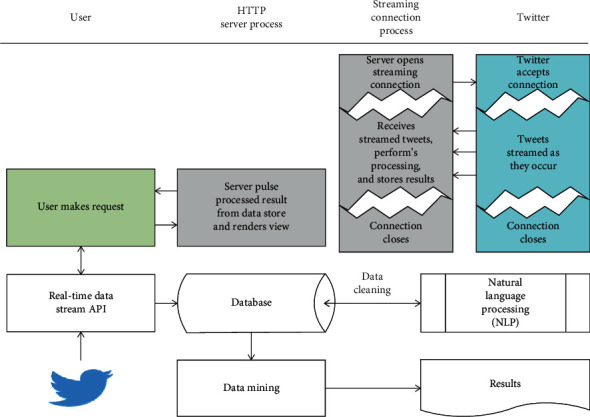
Data collection process via Twitter [28].

As indicated in Figure 1, in the Visual Studio platform by using C# programming language TwitR library, 848 tweets in Turkish with hashtags “#iklimdegisikligi,” “#kureselisinma,” and “#iklimetkisi” are stored in the Microsoft Sql database.

**Figure 2:**
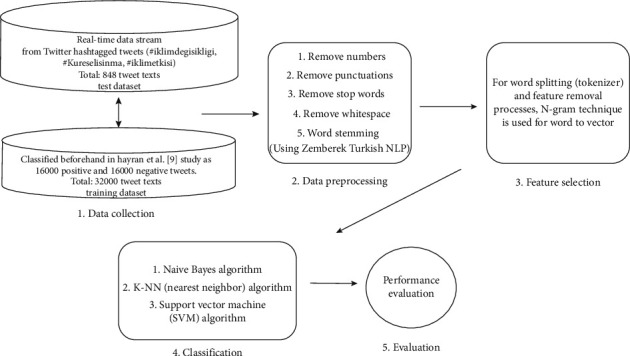
Progress of data evaluation.

In our study, Hayran shared 32 thousand data from his work and had classified content, as a train set after a preoperation. The point that should be emphasized here is that Turkish is a head final language. Therefore, adverbs of time go to the end of the verb in the sentence. In order to minimize semantic shifts and decrease the number of features that would arise, these data are passed through the data preprocessing phase of the 2^nd^ stage in Figure 2. Thus, we have a pure sentence data that is free of punctuation marks and can reduce the semantic shifts. Additionally, since Turkish is a head final language, the adverbs of time are added adjacent to the verb, which will increase our feature number and reduce our chances of successful classification. In order to avoid this, it is aimed to achieve a more effective result by reaching the roots of the words by applying “word stemming” process in Figure 2 and the last step of the 2^nd^ stage with the Turkish NLP library “Zemberek.”

#### 3.1.1. Data Preprocessing

Tweet texts are usually lacking a formal writing standard and because of that each text is purified by implementing the steps in Table 1 to create a sounder model [30, 31]. Purpose of the data preprocessing is to achieve more sensible results by decreasing the size of feature [32–34].

For word stemming, a Turkish NLP library named Zemberek is used. Because of having an MPL licence, general use is allowed. Thanks to this library, after the purification of text after first four steps, roots of the words within the text are determined. After the specified procedures, all data stored in Ms-Sql database are imported to Zemberek library for .Net technology, and then, the word stemming process is implemented. Therefore, the data preprocessing is concluded to achieve a solid NLP process. In addition, data evaluation progress is presented step by step in Figure 2.

## 4. Feature Selection

In this section, numerical equivalents of processed word data are shown, and then, classification methods for emotional analysis are implemented. For word splitting (tokenizer) and feature removal processes, the N-Gram technique is used. It relies on prediction and probability and is studied based upon two main headlines: word and character. In this study, word-based calculation is used. It is described as the probability of a word's position in the sentence related to the preceding word. Gram expresses the weight of the controlled value [19]. In this study, 1–3 is held as constant. According to Markov chain, certain words follow each other frequently, and because of that based on equation (1), it is multiplication of words' conditional probabilities:

(3) $$\begin{array}{c} P\left(w_{1},w_{2}\ldots w_{n}\right)\approx \prod_{i}P\left(w_{i}\left|w_{i-k}\right.\ldots w_{i-1}\right), \end{array}$$

(4) $$\begin{array}{c} P\left(w_{1}\left|w_{1}w_{2}\right.\ldots w_{i-1}\right)\approx P\left(w_{i}\left|w_{i-k}\right.\ldots w_{i-1}\right). \end{array}$$

If we look at each tweet according to equation (4), *P* (global warming problem) = *P* (warming|global)*∗P* (problem | warming), this is how the multiplication of conditioned probabilities is calculated.

## 5. Classification

**Figure 3:**
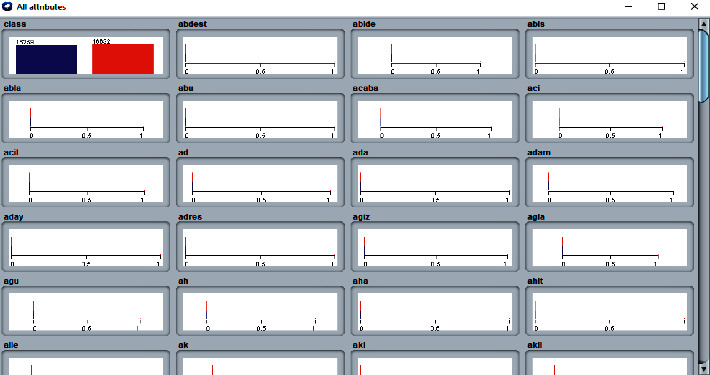
Word variable set.

In the phase of sentiment analysis and classification of tweet data, as the first step, 891 tweets that were pulled from certain hashtags are classified based on emotion (positive is 1 and negative is 0) and separated as test data. As the second step, 16000 positive and 16000 negative tweets are produced and classified beforehand in Hayran et al.'s [35] study and are used as training dataset and attributes, as listed in Table 2 and Figure 3.

Naive Bayes, one of the techniques of supervised machine learning, is subjected to K-NN [24, 36] and SVM classification algorithms [37, 38]. During the procedure of classification, WEKA machine learning tool is used. Used algorithms are explained in the further sections.

Naive Bayes: through the probability procedures implemented within this dataset, classification of the incoming test data is determined, and it is mostly used in word mining classification. Mainly make use of Bayes Rule, *P*(*c|x*) is the posterior probability and *P*(*c|x*) likelihood [39], as shown in the following equations:

(5) $$\begin{array}{c} P\left(c|x\right)=\frac{P\left(x|c\right)P\left(c\right)}{P\left(x\right)}, \end{array}$$

(6) $$\begin{array}{c} P\left(c|x\right)=P\left(x_{1}|c\right)\times P\left(x_{2}|c\right)\times \cdots \times P\left(x_{n}|c\right)\times P\left(c\right). \end{array}$$

K-NN (nearest neighbor): K is used to determine the class of the new data and to store all conditions based upon the distance measure of the nearest neighbor. K-NN is mostly used in pattern recognition and estimation as a nonparametric technique [40]. K value means that how many neighbors should be taken into consideration.

SVM: SVM algorithm is a supervised learning algorithm and binary classifier [25]. It is mostly used to solve classification problems [26]. Support vector machine (SVM) is used to separate data belonging to two classes in a most suitable way, and to implement this procedure, hyperplanes are specified [27].

## 6. Results

### 6.1. Comparative Performance Analysis

Hayran et al. choose the SVM algorithm as the classifier design. They determine the sentiment classification by labelling the texts as a training data. Labelling process is executed manually through using emoji expressions (:), :(, etc.). SVM model is tested with the k-fold cross validation method.

The main reason for the performance value (80.05% accuracy in Table 3) of our study to be lower compared to the work of Hayran et al. is the creation of a training set without removing emotional symbols like smile emotion symbol “:)” and sad emotion symbol “:(” that would significantly affect the classification in their study. If our model has worked hard on our dataset for training in this case, our model starts to memorize. At the same time if our training set is uniform, the risk of overfitting will be high. Therefore, in order to avoid overfitting in our study, an integrated classification method is suggested by removing these sentiment expressions and symbols from our training set. Thus, this is an important factor in model training and successful classification compared to our study.

Erdogan et al. have achieved the highest success rate in their study by making a classification without distinction between Turkish and English text. They used the logistic regression method as a classification tool in their work. Compared to our study, the use of the English dataset and the inclusion of sentimental emoji increased the rate of successful classification. According to similar studies in the table, the logistic regression classifier has been used in four studies. Accuracy results varying between 65% and 94% have been achieved in studies by using this method in Table 3. Ecemis et al. who reached the most successful result have been carried out the classification process using the SVM method. They have performed the classification process by using a manually chosen text set as a training set. Their study presents that, to complete each sentiment class, strong sentimental words in Turkish are used. It has been observed that the selection of sentences containing only adjectives as a training set increases the success rate. Support vector machine classifier used in this study has been preferred as a classification tool in the other three studies in the table. Accuracy results varying between 64% and 80% have been achieved in studies by using this method. In similar studies, it has been observed that SVM, linear regression, and other deep learning methods are mostly preferred as classification tools. The main factor in achieving different performance rates of studies using the same algorithm is the selection of training sets in different structures. It has been observed that the dataset used as a training set increases the success rate of sentences based on certain conditions (emotional symbols and strong sentimental words).

### 6.2. Performance Results

**Figure 4:**
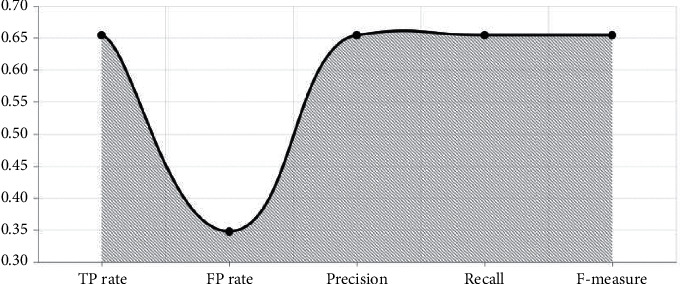
Naive Bayes model evaluation.

**Figure 5:**
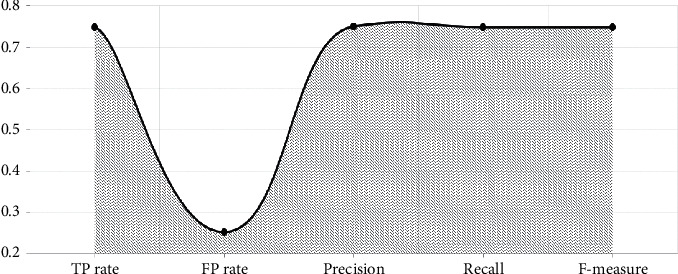
K-NN (nearest neighbor) model evaluation.

**Figure 6:**
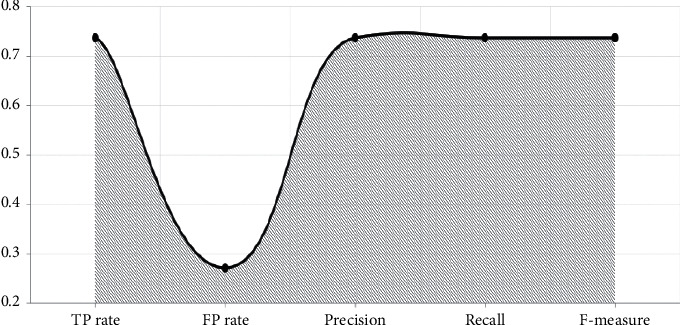
Support vector machine (SVM) model evaluation.

With the established model in Figures 4–6, performance measure comparison for the dataset subjected to classification algorithms via WEKA is shown in Table 4 according to the evaluation measures. In Table 4, it is shown that K-NN algorithm is more successful than others. We reached as a result 74.63 percent accuracy on this enhanced algorithm. In the text preprocessing, using “Word to Vector” as “*n*-gram” algorithm and taking advantage of Zemberek library increased rates of success to find word roots. We use the evaluations metrics which are precision, sensitivity, F-measure, and accuracy [55, 56]. These metrics are depending on TP (true-positive) and FP (false-positive) ratios:

(7) $$\begin{array}{c} \text{precision}=\frac{\text{TP}}{\left(\text{TP}+\text{FP}\right)}, \end{array}$$

(8) $$\begin{array}{c} \text{sensitivity}=\frac{\text{TP}}{P}=\frac{\text{TP}}{\left(\text{TP}+\text{FN}\right)}, \end{array}$$

(9) $$\begin{array}{c} F-\text{measure }=\frac{2\text{TP}}{\left(2\text{TP }+\text{ FP }+\text{FN}\right)}, \end{array}$$

(10) $$\begin{array}{c} \text{accuracy }=\frac{\left(\text{TP }+\text{TN}\right)}{\left(P+N\right)}. \end{array}$$

In the case of reducing the number of variables and achieving more successful results, Zemberek is used as the Natural Language Processing library to find the roots in each Turkish tweet. Since each word in the sentence does not always make sense alone, vectors are created by using the *N*-gram (2, 3) technique that treats words in dual and triple groups. In order to find out whether this integrated technique is successful, testing has been conducted on three different classification algorithms.

**Figure 7:**
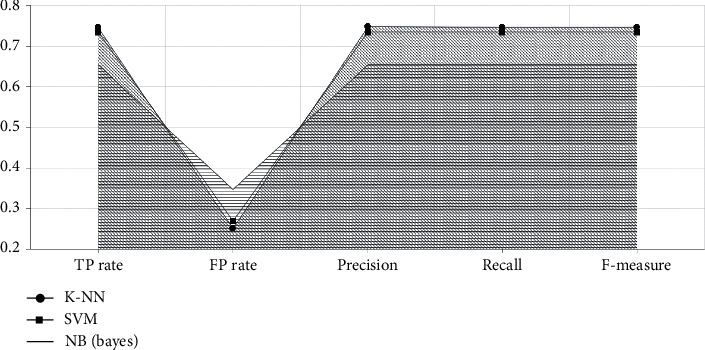
Metric comparison of models.

The results of the proposed method have been tested on the Turkish tweet dataset, and the highest performance rate has been obtained with the K-NN classification algorithm used with integrated technique in Table 4. Compared to other classification algorithms in Figure 7, the highest success rate (74.63%) has been achieved by using the K-NN classification algorithm with the N-gram technique because the K-NN classification algorithm classifies on the basis of the closest neighbor proximity as the principle of operation. Therefore, with the N-gram (2.3) technique that creates vectors by considering the frequency of using words together, more successful results are obtained compared to other classification algorithms in Table 5.

## 7. Conclusion

With the growth of social media in recent years, it has become an important research resource for people's ideas on specific issues. Accordingly, emotion analysis on texts with social media data has been the subject of research. It is important for forward-looking plans with measures and actions to make an emotion detection on subject. We summarized the results of the comparative methods for the analysis of social sensitivity and mentioned promising aspects in this field. The dataset used to support the findings of this study has been deposited in the “Sentiment Analysis on Turkish Tweets Dataset” repository on online data library [57, 58].

In this study, posted tweets about climate change, which is one of the biggest environmental topics, associated with this attempted to establish automatically emotion analysis. Therefore, a way is clear to ascertain public opinion and precautions about environmental topics by emotion analysis. We observed that using integrated classification methods instead of a single machine learning technique increased the success rate of accuracy. Considering the high rate of double or triple word groups in Turkish language, it is recommended to choose this integrated method in emotion classification studies. Using word splitting (tokenizer) in the phase of data preprocessing, “Zemberek” library for finding word roots and recommended integrated solution as N-gram for the feature removal process by using K-NN classification machine learning algorithm increased success rates of text analysis, in the case of especially texts in Turkish language.

## Figures and Tables

**Table 1:** Data preprocessing steps.

Remove numbers	Deleting numerical expressions in the texts
Remove punctuations	Deleting special characters and punctuation marks in the texts
Remove stop words	Removal of stop words that do not change the meaning of the sentence specified for Turkish
Remove whitespace	Deleting the blank characters in the text
Word stemming	Determining the word roots using Zemberek Turkish NLP in the sentence [29]

**Table 2:** Training set attributes.

@Relation train
@attribute document string
@attribute sentiment class {1,0}
@data

**Table 3:** Emotion analysis studies in Turkish language.

Authors	Methodology	Data	Indicators	Performance result
Erdogan et al. [41]	*n*-gram (1, 2, 3) method, logistic regression	2018	Five most used cryptocurrencies in English text tweets	94.60
Ciftci et al. [42]	RNN-based algorithm	2018	Turkish Wikipedia articles	83.30
Coban et al. [43]	BoW vs W2VC model	2013	Turkish Twitter messages in the telecom sector	59.17
Ecemiş et al. [44]	Support vector machine	2018	Turkey-based geographical user data	0.954
Isik et al. [45]	Novel stacked ensemble method for sentiment analysis	2018	IMDB dataset including 1000 positive and 1000 negative; 2000 movie comments have been used	0.791
Karcioglu et al. [46]	Linear SVM and logistics regression	2019	Random English and Turkish texts have been collected by Twitter	65.62
Uslu et al. [47]	Logistics regression	2019	User reviews have been collected from Turkey's most preferred movie site	77.35
Kanmaz et al. [48]	Decision trees, support vector machine, and Naive Bayes methods	1996–2018	News text-related stock exchange	0.64–0.80
Doğan et al. [49]	LSTM recurrent neural networks	2019	In the study, a single mixed data pool with two categories is created with data collected from multiple social networks	0.9194–0.9266
Salur et al. [50]	Random forest classification method	2019	Tweets collected about special tourism centers	88.974
Santur [51]	Gated recurrent unit method	2019	Turkish e-commerce platform user reviews	0.955
Kamis et al. [52]	Multiple CNN's and LSTM network	2017	A corpus of different datasets is utilized based on three datasets used in SemEval (semantic assessment)	0.59
Ogul et al. [53]	Logistic regression classifier	2017	Public SemEval (semantic assessment) in three different sentiment analysis datasets containing both Turkish and English texts	79.56
Rumelli et al. [54]	*k*-nearest neighbor classifier	2019	The dataset is built by using e-commerce website (http://www.hepsiburada.com); the user review, rating, and URL of the product have been analyzed	73.8
Hayran et al. [35]	Support vector machine (SVM) classifier	2017	A Turkish text dataset classified (16000 positive and 16000 negative emotion) by emoji icon	80.05

**Table 4:** Evaluation results.

Classifier	TP rate	FP rate	Precision	Recall	F-Measure
K-NN	0.746	0.251	0.748	0.746	0.746
SVM	0.735	0.269	0.735	0.735	0.735
NB (Bayes)	0.654	0.347	0.654	0.654	0.654

**Table 5:** Recommended combined technique.

Integrated technique	Classification algorithm	Accuracy (%)
Zemberek Turkish NLP (word stemming), N-gram (2.3)	K-NN	74.63
Zemberek Turkish NLP (word stemming), N-gram (2.3)	SVM	73.51
Zemberek Turkish NLP (word stemming), N-gram (2.3)	NB (Bayes)	65.43

## Data Availability

All the raw data will be made available if needed.

## References

[1] IPCC (2014). *Climate Change 2014: Synthesis Report*.

[2] Pourebrahim N., Sultana S., Edwards J., Gochanour A., Mohanty S. (2019). Understanding communication dynamics on twitter during natural disasters: a case study of Hurricane Sandy. *International Journal of Disaster Risk Reduction*.

[3] Yang W., Mu L., Shen Y. (2015). Effect of climate and seasonality on depressed mood among twitter users. *Applied Geography*.

[4] Roxburgh N., Guan D., Shin K. J. (2019). Characterising climate change discourse on social media during extreme weather events. *Global Environmental Change*.

[5] Abbot J., Marohasy J. (2017). The application of machine learning for evaluating anthropogenic versus natural climate change. *GeoResJ*.

[6] Gümüşçü A., Tenekeci M. E., Bilgili A. V. (2019). Estimation of wheat planting date using machine learning algorithms based on available climate data. *Sustainable Computing: Informatics and Systems*.

[7] Kemer E., Samli R. (2019). Performance comparison of scalable rest application programming interfaces in different platforms. *Computer Standards & Interfaces*.

[8] Li J., Li N., Afsari K., Peng J., Wu Z., Cui H. (2019). Integration of building information modeling and web service application programming interface for assessing building surroundings in early design stages. *Building and Environment*.

[9] Lago D., Rahnema F. (2017). Development of an application programming interface for depletion analysis (APIDA). *Annals of Nuclear Energy*.

[10] Statista. [Online]

[11] Dragoni M., Petrucci G. (2018). A fuzzy-based strategy for multi-domain sentiment analysis. *International Journal of Approximate Reasoning*.

[12] Amplayo R. K., Lee S., Song M. (2018). Incorporating product description to sentiment topic models for improved aspect-based sentiment analysis. *Information Sciences*.

[13] Sokhin T., Butakov N. (2018). Semi-automatic sentiment analysis based on topic modeling. *Procedia Computer Science*.

[14] Ruan Y., Durresi A., Alfantoukh L. (2018). Using twitter trust network for stock market analysis. *Knowledge-Based Systems*.

[15] Piro P., Nock R., Nielsen F., Barlaud M. (2012). Leveraging k-NN for generic classification boosting. *Neurocomputing*.

[16] Zhang F., Wang C., Yang F. (2019). Pattern-based NN control for uncertain pure-feedback nonlinear systems. *Journal of the Franklin Institute*.

[17] Chen Y., Zhou Y. (2020). Machine learning based decision making for time varying systems: parameter estimation and performance optimization. *Knowledge-Based Systems*.

[18] Song C., Wang X.-K., Cheng P.-F., Wang J.-Q., Li L. (2020). SACPC: a framework based on probabilistic linguistic terms for short text sentiment analysis. *Knowledge-Based Systems*.

[19] Dey A., Jenamani M., Thakkar J. J. (2018). Senti-N-Gram : an *n*-gram lexicon for sentiment analysis. *Expert Systems with Applications*.

[20] Pang B., Lee L., Vaithyanathan S. Thumbs up?.

[21] Kaur J., Sehra S. S., Sehra S. K. (2016). Sentiment analysis of twitter data using hybrid method of support vector machine and ant colony optimization. *International Journal of Computer Science and Information Security (IJCSIS)*.

[22] Taboada M., Brooke J., Tofiloski M., Voll K., Stede M. (2011). Lexicon-based methods for sentiment analysis. *Computational Linguistics*.

[23] Kaya M., Fydan G., Toroslu I. Sentiment analysis of Turkish political news.

[24] Gómez P., Partal A., Espinilla M. (2017). Classification of the risk in the new financing framework of the deposit guarantee systems in europe: K-means cluster Analysis and soft computing. *International Journal of Computational Intelligence Systems*.

[25] Güraksın G. E., Uğuz H. (2018). Comparison of different training data reduction approaches for fast support vector machines based on principal component analysis and distance based measurements. *International Journal of Computational and Experimental Science and Engineering*.

[26] Yüksel A. S., Çankaya Ş. F., Üncü İ. S. (2017). Design of a machine learning based predictive analytics system for spam problem. *Acta Physica Polonica A*.

[27] Ramesh B., Sathiaseelan J. G. R. (2015). An advanced multi class instance selection based support vector machine for text classification. *Procedia Computer Science*.

[28] Savas S., Topaloglu N. Crime intelligence from social media: a case study.

[29] Zemberek-NLP

[30] Sharma A. K., Yadav R. Spam mails filtering using different classifiers with feature selection and reduction technique.

[31] Azimi P., Soofi P. (2017). An ANN-based optimization model for facility layout problem using simulation technique. *Scientia Iranica*.

[32] Kamisli Ozturk Z., Erzurum Cicek Z. İ., Ergul Z. (2017). Sentiment analysis: an application to anadolu university. *Acta Physica Polonica A*.

[33] Rabbani M., Habibnejad-Ledari H., Rafiei H., Farshbaf-Geranmayeh A. (2016). A bi-objective mathematical model for dynamic cell formation problem considering learning eect, human issues, and worker assignment. *Scientia Iranica*.

[34] Nazari L., Seifbarghy M., Setak M. (2017). Modeling and analyzing pricing and inventory problem in a closed loop supply chain with return policy and multiple manufacturers and multiple sales channels using game theory. *Scientia Iranica*.

[35] Hayran A., Sert M. Sentiment analysis on microblog data based on word embedding and fusion techniques.

[36] Liu F., Wang T., Guan S.-U., Man K. L. (2015). Neural incremental attribute learning in groups. *International Journal of Computational Intelligence Systems*.

[37] Nourmohammadi Khiarak J., Vali̇zadeh-kamran R., Heydariyan A., Damghani N., Damghani N. (2018). Big data analysis in plant science and machine learning tool applications in genomics and proteomics. *International Journal of Computational and Experimental Science and Engineering*.

[38] Abubakar A., Chiroma H., Zeki A., Uddin M. (2016). Utilising key climate element variability for the prediction of future climate change using a support vector machine model. *International Journal of Global Warming*.

[39] Jiang L., Li C., Wang S., Zhang L. (2016). Deep feature weighting for naive Bayes and its application to text classification. *Engineering Applications of Artificial Intelligence*.

[40] Tan S. (2006). An effective refinement strategy for KNN text classifier. *Expert Systems with Applications*.

[41] Erdoğan M. C., Canayaz M. Crypto-currency sentiment analyse on social media.

[42] Ciftci B., Apaydin M. S. A deep learning approach to sentiment analysis in Turkish.

[43] Çoban Ö., Özyer G. T. Word2vec and clustering based twitter sentiment analysis.

[44] Ecemiş A., Dokuz A. Ş., Çelik M. Sentiment analysis of posts of social media users in their socially important locations.

[45] Emre Isik Y., Görmez Y., Kaynar O., Aydin Z. NSEM: novel stacked ensemble method for sentiment analysis.

[46] Karcioğlu A. A., Aydin T. Sentiment analysis of Turkish and english twitter feeds using Word2Vec model.

[47] Uslu A., Tekin S., Aytekin T. Sentiment analysis in Turkish film comments.

[48] Kanmaz M., Surer E. Positive or negative? a semantic orientation of financial news.

[49] Doğan E., Kaya B. Deep learning based sentiment analysis and text summarization in social networks.

[50] Salur M. U., Aydin İ., Alghrsi S. A. SmartSenti: a twitter-based sentiment analysis system for the smart tourism in Turkey.

[51] Santur Y. Sentiment analysis based on gated recurrent unit.

[52] Goularas D., Kamis S. Evaluation of deep learning techniques in sentiment analysis from twitter data.

[53] Oğul H. A., Güran A. Imbalanced dataset problem in sentiment analysis.

[54] Rumelli M., Akkuş D., Kart Ö., Isik Z. Sentiment analysis in Turkish text with machine learning algorithms.

[55] Cömert Z., Kocamaz A. F. (2017). Comparison of machine learning techniques for fetal heart rate classification. *Acta Physica Polonica A*.

[56] Navazi A., Karbassi A., Mohammadi S., Monavari S. M., Zarandi S. M. (2017). A modelling study for predicting temperature and precipitation variations. *International Journal of Global Warming*.

[57] Kırelli Y., Arslankaya S. (2020). *Sentiment Analysis on Turkish Tweets Dataset, Dryad, Dataset*.

[58] Kırelli Y., Arslankaya S. (2020).

